# A Hybrid Missing Data Imputation Method for Batch Process Monitoring Dataset

**DOI:** 10.3390/s23218678

**Published:** 2023-10-24

**Authors:** Qihong Gan, Lang Gong, Dasha Hu, Yuming Jiang, Xuefeng Ding

**Affiliations:** 1 Informatization Construction and Management Office, Sichuan University, Chengdu 610065, China; gqh@scu.edu.cn; 2 Big Data Analysis and Fusion Application Technology Engineering Laboratory of Sichuan Province, Chengdu 610065, China; 2019223049250@stu.scu.edu.cn (L.G.); hudasha@scu.edu.cn (D.H.); jiangym@scu.edu.cn (Y.J.); 3 College of Computer Science, Sichuan University, Chengdu 610065, China

**Keywords:** batch process, data quality, missing data imputation, LSTM neural network

## Abstract

Batch process monitoring datasets usually contain missing data, which decreases the performance of data-driven modeling for fault identification and optimal control. Many methods have been proposed to impute missing data; however, they do not fulfill the need for data quality, especially in sensor datasets with different types of missing data. We propose a hybrid missing data imputation method for batch process monitoring datasets with multi-type missing data. In this method, the missing data is first classified into five categories based on the continuous missing duration and the number of variables missing simultaneously. Then, different categories of missing data are step-by-step imputed considering their unique characteristics. A combination of three single-dimensional interpolation models is employed to impute transient isolated missing values. An iterative imputation based on a multivariate regression model is designed for imputing long-term missing variables, and a combination model based on single-dimensional interpolation and multivariate regression is proposed for imputing short-term missing variables. The Long Short-Term Memory (LSTM) model is utilized to impute both short-term and long-term missing samples. Finally, a series of experiments for different categories of missing data were conducted based on a real-world batch process monitoring dataset. The results demonstrate that the proposed method achieves higher imputation accuracy than other comparative methods.

## 1. Introduction

The batch process is an important production mode in the modern manufacturing industry. As a highly flexible production method, the batch process is essential in producing low-volume, high-value-added products, such as chemical and biological materials [1,2]. With the rapid development of the Internet of Things and sensing technology [3], the monitoring data of batch processes is being recorded more frequently. However, batch process monitoring data often contains missing values due to factors such as external environmental conditions, link failures, and sensor equipment degradation. This results in incomplete and unreliable batch process monitoring data, which poses a significant obstacle to the subsequent utilization of the data [4]. Especially, missing data will decrease the performance of data-driven modeling for fault identification and optimal control in batch processes. Therefore, it is significant to study how to deal with missing data to enhance the quality of batch process monitoring data.

There are mainly two categories of methods to handle missing data: deletion and imputation [5,6]. The deletion method may not only lose valuable information within the data but also destroy the continuity of the time series, leading to inaccurate results in subsequent data analysis. The imputation method involves replacing missing values with predicted values [7], which is more suitable for improving data quality. However, there are few studies that focus on missing data imputation for batch process monitoring datasets. Nomikos et al. [8] employed the mean method for imputing missing values. Laila et al. [9] and Meng et al. [10] introduced a methodology where the unknown observations are calculated using a weighted combination of scores from the current time point in the new batch and previously computed scores from a calibration dataset. Shi et al. [11] established a linear regression model that uses several historical values adjacent to the current time to predict the missing values. Further research is needed, as the imputation results of these methods have shown limited effectiveness.

Due to the characteristics of batch processes, such as multiple operating conditions, multiple batches, and multiple stages, missing data in batch process monitoring datasets usually presents a complex situation, making it challenging to perform accurate imputation. Furthermore, batch process monitoring datasets contain different types of missing data and directly applying an existing single method cannot achieve favorable imputation results. Consequently, how to combine or improve appropriate imputation models to effectively impute missing data within batch process monitoring datasets is still a significant problem to be solved.

In this paper, we propose a hybrid missing data imputation method for batch process monitoring datasets based on single-dimensional interpolation, a multivariate regression model, and LSTM. The main contributions are as follows:

- We propose a missing data classification method based on the continuous missing duration for each variable and the number of variables missing simultaneously. Then we classify the missing data into five distinct categories: transient isolated missing values, short-term missing variables, long-term missing variables, short-term missing samples, and long-term missing samples.
- We design and implement the hybrid missing data imputation method to deal with different categories of missing data step by step, taking into account the characteristics of different categories of missing data. This method employs a combination of three single-dimensional interpolation models that enables the automated detection and imputation of transient isolated missing values. We design an iterative imputation based on a multivariate regression model to automatically complete the imputation of all long-term missing variables. To address short-term missing variables, we propose a combination model based on single-dimensional interpolation and multivariate regression by utilizing system fluctuations. We use the LSTM model to impute both short-term and long-term missing samples.
- We have carried out extensive experiments on a real-world injection molding process monitoring dataset to demonstrate the effectiveness and accuracy of the proposed hybrid missing data imputation method.

The remainder of this paper is structured as follows. Section 2 presents the related works. Section 3 describes the hybrid missing data imputation method designed. Section 4 verifies the validity of the proposed method by taking a real-world injection molding process monitoring dataset as an example. Section 5 presents the conclusions.

## 2. Related Works

Many imputation techniques have been proposed for different domain-specific datasets [12], primarily involving two categories: statistical and machine learning-based techniques [13,14].

Statistical imputation techniques rely on statistical models to predict missing values. Simple imputation handles missing values by using methods such as the mode, mean, or median of the available values [15]. Hot-deck imputation handles missing values by replacing them with similar object values [16]. Interpolation methods, which mainly include nearest neighbor interpolation, linear interpolation, and spline interpolation, estimate missing values by establishing interpolation functions [17]. These techniques perform imputation based on temporal continuity and are effective in the case of a handful of missing values. Regression imputation involves estimating relationships among variables using regression modeling [18], which typically includes Linear Regression (LR) and Multivariate Linear Regression (MLR). This approach can effectively utilize the correlations between time series data for imputation. Matrix-based methods recover missing data by treating an entire set of series as a matrix and applying techniques based on matrix completion principles [19]. These techniques leverage temporal continuity for imputation and mainly include Singular Value Decomposition (SVD), Principal Component Analysis (PCA), Matrix Factorization (MF), and Centroid Decomposition (CD)-based methods. PCA-based methods, SPIRIT [20] and ROSL [21], are effective for datasets with a limited number of time series or short time series. SVD-based SoftImpute [22] and MF-based TRMF [23] require data to contain repeating trends, while CD-based CDRec [24] is only effective for correlated time series. Pattern-based methods utilize pattern-matching techniques for imputation by leveraging trend similarity. For instance, STMVL [25] derives statistical models from historical data and requires highly correlated time series. DynaMMo [26] employs Kalman filters and Expectation-Maximization (EM) for imputation and is adaptable to datasets with irregular fluctuations.

Machine learning techniques are widely used in various practical application fields, such as air pollution monitoring [27], industrial process monitoring [2], dam safety monitoring [28,29], medical data processing [30], and stock price prediction [31]. To address the challenges posed by missing data, several machine learning-based methods have gained significant popularity [12]. The K Nearest Neighbor (KNN) algorithm [32] works by classifying the nearest neighbors of missing values and using those neighbors for imputation through a distance measure between instances. The Random Forest (RF) algorithm [33,34] constructs multiple decision trees based on the bootstrapping procedure and gives the final predictions by the averaged values or majority votes of each tree’s prediction. The K-means clustering algorithm [35] consists of 2 steps, where the first step gets clusters using K-means clustering, and then the second step handles missing values using cluster information. These methods utilize the correlation between time series but do not consider the continuity in the time dimension. And more advanced neural networks have also been applied to deal with missing values in time series data. The Extreme Learning Machine (ELM) [36] is an efficient machine learning model based on a single-layer feedforward neural network and is suitable for multi-dimensional time series with multiple features. Long Short-Term Memory (LSTM) [37], which is an improved form of Recurrent Neural Networks (RNNs) [38], can effectively learn long-term dependencies for predicting multi-dimensional time series.

In summary, although several imputation methods have been proposed, most of them are typically designed to estimate a specific type of missing data. And these methods often excel only when handling datasets with specific data characteristics. In practical domains, such as batch process monitoring datasets, missing data usually presents a complex situation. These datasets contain different types of missing data, and different types of missing data exhibit distinct characteristics. Applying a single imputation method directly may not be effective. Therefore, further research is still needed on how to conduct classification analysis of missing data and design a hybrid method by employing suitable imputation techniques tailored to the characteristics of different types of missing data.

## 3. Methodology

### 3.1. Data Processing

#### 3.1.1. Data Unfolding

**Figure 1:**
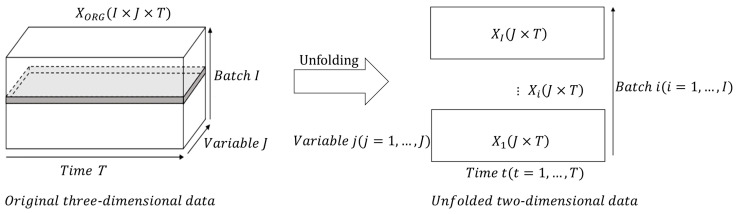
Unfolding data along the batch dimension.

For a typical batch process, the monitoring data is stored in a three-dimensional matrix, $X_{ORG}(I\times J\times T)$, where *I* represents the number of batches, *J* represents the number of process variables, and *T* represents the number of sampling moments in a batch. Since subsequent research on missing data imputation involves analyzing and processing missing variables at different sampling moments, it is necessary to unfold the original three-dimensional data along the batch dimension to obtain two-dimensional data, that is, $X_{i}\left(J\times T\right)$ of i(i=1, …, I) batches. As shown in Figure 1, *I* matrix slices are obtained by unfolding the original three-dimensional data along the batch dimension. Each matrix slice represents a set of values for variable j(j=1, …, J) at sampling moments t(t=1, …, T).

#### 3.1.2. Missing Data Classifying

The assumption in this paper is to impute missing data based on dataset denoising. The missing data can arise from data acquisition as well as from data denoising. Regarding the missing data caused by data acquisition, the causes of missing data in batch process monitoring can be summarized into the following three cases: (1) Production equipment outage, acquisition system failures, or data link failures lead to long or short periods of continuous missing for many variables; (2) Acquisition equipment failures lead to long or short periods of continuous missing for a few variables; (3) The instability or aging of acquisition equipment leads to isolated missing values for a few variables.

Based on the cause analysis of missing data, the classification rules for missing data are defined, as shown in Table 1. ∆t represents the continuous missing duration of a variable, $n_{v}$ represents the number of variables missing simultaneously during this period. $T_{0}$ represents the data sampling interval, ${Th}_{t1}$ represents the time threshold at which the data trend does not change, ${Th}_{t2}$ represents the time threshold at which the data trend can be predicted. ${Th}_{t1}$ and ${Th}_{t2}$ are set according to the specific situation of different variables and the practical requirements for data analysis. Variable threshold ${Th}_{v}$ represents the critical value for the number of variables missing simultaneously in a certain period (longer than ${Th}_{t1}$), and ${Th}_{v}$ is set to ⌊n/2⌋, where n represents the number of variables in batch process monitoring dataset.

By calculating the continuous missing duration ∆t for each variable and the corresponding number of variables $n_{v}$ missing simultaneously, and then comparing the calculated results with the threshold values, the missing data is classified into five categories: transient isolated missing values, short-term missing variables, long-term missing variables, short-term missing samples, and long-term missing samples. Short-term and long-term missing variables are categorized as continuous missing variables, while short-term and long-term missing samples are categorized as continuous missing samples. Variables without any missing values are referred to as complete variables, while variables with missing values are referred to as incomplete variables.

### 3.2. Missing Data Imputation

#### 3.2.1. Dataset Splitting

Due to the presence of many incomplete variables within the continuous missing samples, it can be considered that a system outage occurred during this period. The data segment with continuous missing samples can be seen as a missing data segment. Therefore, the unfolded dataset needs to be split into several data segments according to the locations of continuous missing samples and then imputed. Assuming that the dataset *X* is split K−1 times, then the dataset *X* contains *K* data segments and K−1 missing data segments (data segments with short-term or long-term missing samples):

(1) $$X={\left[X_{1},X_{1}^{*},\ldots ,X_{k},X_{k^{*}}^{*}{,\ldots ,\;X_{K-1},X}_{K-1}^{*},\;X_{K}\;\right]}^{T}$$

where $X_{k}(k=1,\;...,\;K)$ represent the *k*-th data segment, and each data segment $X_{k}$ contains only transient isolated missing values, short-term or long-term missing variables, $X_{k^{*}}^{*}(k^{*}=1,\;...,\;K-1)$ represent the missing data segment between the *k*-th and (*k +* 1)-th data segments.

Variable Missing Proportion (VMP) and Sample Missing Proportion (SMP) are introduced as measures to describe the extent of missing data within each data segment. Taking data segment $X_{k}\in R^{mk\times n}$ as an example, the sample missing proportion ${SMP}_{k}$ of $X_{k}$ and the variable missing proportion ${VMP}_{k\_j}$ of variable j in $X_{k}$ are calculated as follows:

(2) $${SMP}_{k}=1-m_{int\_k}/mk\;{VMP}_{k\_j}=1-m_{int\_k\_j}/mk$$

where mk is the sample size of $X_{k}$, *n* is the number of variables in $X_{k}$, $m_{int\_k}$ represents the sample size without missing values, and $m_{int\_k\_j}$ represents the number of values that are not missing in variable *j*.

#### 3.2.2. Transient Isolated Missing Values Imputation

For transient isolated missing values, the data trend in the time dimension remains unchanged. The missing values can be estimated using single-dimensional interpolation models based on temporal continuity. The nearest neighbor interpolation, linear interpolation and cubic spline interpolation are used. Assuming that $x_{i,j}$ (the *i*-th value of variable *j*) in data segment $X_{k}$ is missing, and ${\tilde{x}}_{i,j}$ represents the estimated value of $x_{i,j}$.

(1) Single-dimensional Interpolation Model

The nearest neighbor interpolation: The interpolation function is established using a valid value adjacent to $x_{i,j}$, as shown in Formula (3). The limitation of this method is the discontinuity at ${\tilde{x}}_{i,j}$.

(3) $${\tilde{x}}_{i,j}=x_{i-1,j}\;\left(or=x_{i+1,j}\right)$$

The linear interpolation: The interpolation function is constructed using two valid value adjacent to $x_{i,j}$, as shown in Formula (4). While linear interpolation ensures continuity at ${\tilde{x}}_{i,j}$, it lacks derivability at the endpoints.

(4) $${\tilde{x}}_{i,j}=\frac{1}{2}(x_{i-1,j}+x_{i+1,j})$$

The cubic spline interpolation: The cubic spline interpolation requires at least four valid values and constructs the interpolation function using two adjacent values before $x_{i,j}$ and two adjacent values after $x_{i,j}$, as shown in Formula (5). The detailed construction process can be found in reference [39].

(5) $${\tilde{x}}_{i,j}=f_{spline}(x_{i-2,j},x_{i-1,j},x_{i+1,j},x_{i+2,j})$$

When both values $x_{i,j}$ and $x_{i+1,j}$ are missing simultaneously (${Th}_{t1}$ is set to 2), the interpolation Formulas (3), (4), and (5) need to be reconstructed, respectively, as shown in Formulas (6)–(8).

(6) $${\tilde{x}}_{i,j}={\tilde{x}}_{i+1,j}=x_{i-1,j}\left(or=x_{i+2,j}\right)$$

(7) $$\left\{\begin{array}{c} {\tilde{x}}_{i,j}=x_{i-1,j}+\frac{1}{3}(x_{i+2,j}-x_{i-1,j})\\ {\tilde{x}}_{i+1,j}=x_{i-1,j}+\frac{2}{3}(x_{i+2,j}-x_{i-1,j}) \end{array}\right.$$

(8) $$\left\{\begin{array}{c} {\tilde{x}}_{i,j}=f_{spline}^{\left(i\right)}\left(x_{i-2,j},\;x_{i-1,j},\;x_{i+2,j},x_{i+3,j}\right)\\ {\tilde{x}}_{i+1,j}=f_{spline}^{\left(i+1\right)}\left(x_{i-2,j},\;x_{i-1,j},\;x_{i+2,j},x_{i+3,j}\right) \end{array}\right.$$

where ${\tilde{x}}_{i+1,j}$ is the interpolated value of $x_{i+1,j}$, $f_{spline}^{\left(i\right)}$ and $f_{spline}^{\left(i+1\right)}$, respectively, represent the cubic spline interpolation functions for $x_{i,j}$ and $x_{i+1,j}$.

(2) Imputation Process for Transient Isolated Missing Values

To impute the transient isolated missing values $x_{i,j}$ in the data segment $X_{k}$, a combination of the above three interpolation models is employed. Combining these three methods enables the automated detection and imputation of transient isolated missing values, making it an efficient complementary approach. When four adjacent valid values are available, cubic spline interpolation is utilized for imputation. If the four adjacent values do not consist of two values before $x_{i,j}$ and two values after $x_{i,j}$, the cubic spline interpolation function needs to be adjusted. Taking one value before $x_{i,j}$ and three values after $x_{i,j}$ as an example, the adjusted cubic spline interpolation function is shown in Formula (9).

(9) $${\tilde{x}}_{i,j}=f_{spline}^{\left(i\right)}\left(x_{i-1,j},x_{i+1,j},x_{i+2,j},x_{i+3,j}\right)$$

When the missing value is located at the endpoint of $X_{k}$, meaning that only one side (either left or right) has an adjacent value, the nearest neighbor interpolation is utilized for imputation. When two adjacent valid values are available, with one before and one after $x_{i,j}$, the linear interpolation is used for imputation.

#### 3.2.3. Continuous Missing Variables Imputation

In the case of a long-term missing variable, significant information in the time dimension is seriously lost. The missing values of the long-term missing variable can only be estimated based on the correlation with other complete variables. The multivariate regression model is suitable for imputing missing values for long-term missing variables. The model constructs a regression function between the long-term missing variable and other complete variables based on their correlations. Then, by utilizing the complete variables as input, the missing values of the long-term missing variable can be predicted. In the case of a short-term missing variable, the missing values can be estimated by considering the correlation with other complete variables, together with the data trend in the time dimension. Therefore, a combination model based on single-dimensional interpolation and multivariate regression is proposed to impute the missing values of short-term missing variables by combining the strengths of both models.

(1) Multivariate Regression Model

Three widely used multivariate regression models are chosen for this study: MLR, RF, and KNN. All three models exhibit robustness and require minimal or no parameters. Assuming that $X_{train}\in R^{mt\times n}$ and $Y_{train}\in R^{mt\times 1}$ are the input and output of training data, respectively, and $X_{test}\in R^{ms\times n}$ and $Y_{test}\in R^{ms\times 1}$ are the input and output of testing data, respectively, where mt represents the sample size of the training data, *n* represents the number of variables, ms represents the sample size of the testing data.

MLR establishes a linear regression function by considering the correlation between the incomplete variable and other complete variables. Then, the function is utilized to predict the missing values. An advantage of the MLR model is its lack of reliance on hyperparameters. The missing values imputation process using MLR is as follows:

Step 1: Modeling. Construct the MLR function:

(10) $$Y_{train}=X_{D}\theta +\varepsilon$$

where $X_{D}$ is the design matrix for $X_{train}$ and $X_{D}=\left[I_{train},X_{train}\right]$, $I_{train}={\left[1,...,1\right]}^{T}\in R^{mt\times 1}$ is a constant vector, $\varepsilon ={\left[\varepsilon_{0},\varepsilon_{1},\ldots ,\varepsilon_{m}\right]}^{T}\in R^{mt\times 1}$ is the error vector, $\theta ={\left[\theta_{0},\theta_{1},...,\theta_{n}\right]}^{T}\in R^{(n+1)\times 1}$ is the coefficient vector, θ can be estimated by Formula (11):

(11) $$\tilde{\theta }={\left(X_{D}^{T}X_{D}\right)}^{-1}X_{D}^{T}Y_{train}$$

where $\tilde{\theta }$ is the estimated value of θ, $X_{D}^{T}$ is the transpose matrix of $X_{D}$, ${\left(X_{D}^{T}X_{D}\right)}^{-1}$ is the inverse matrix of $X_{D}^{T}$ and $X_{D}$.

Step 2: Missing values prediction. Estimate $Y_{test}$ using $X_{test}$:

(12) $$Y_{test}=X_{P}\tilde{\theta }$$

where $X_{P}$ is the design matrix for $X_{test}$ and $X_{P}=\left[I_{test},\;X_{test}\right]$, $I_{test}={\left[1,...,1\right]}^{T}\in R^{ms\times 1}$ is a constant vector.

RF is an ensemble learning model based on the Classification and Regression Tree (CART). The RF model requires two hyperparameters n_estimators and m_features, which respectively represent the number of trees and the number of selected features. The missing value imputation process using the RF model is as follows:

Step 1: RF model training.

Step 1.1: Utilize the Bootstrap resampling method to select n_estimators samples from the original training dataset with replacement, and remove duplicate samples to create a new training dataset $D_{t}=\left\{X_{train}\left(1\right),Y_{train}\left(1\right)\right\}$.

Step 1.2: Train CART decision trees using dataset $D_{t}$ to generate the trained CART model $CART\_model\left(1\right)$. During the training process, randomly select m_features features from all the features, and then identify the optimal feature within the selected features as the splitting point for partitioning each node into left and right segments.

Step 1.3: Repeat Steps 1.1–1.2 n_estimators times to obtain n_estimators CART decision trees, denoted as the prediction model $\left\{CART\_model\right\}$.

Step 2: Missing values prediction.

Step 2.1: Select the same m_features features as used in the training process to create a new testing dataset $X_{test}(1)$.

Step 2.2: Input $X_{test}(1)$ into the trained model $\left\{CART\_model\;\left(1\right)\right\}$ to obtain the first prediction result $Y_{test}(1)$.

Step 2.3: Repeat Steps 2.1–2.2 until obtaining n_estimators prediction results.

Step 2.4: Calculate the final prediction result $Y_{test}$ using the mean method:

(13) $$Y_{test}=\frac{1}{n\_estimators}\times \sum_{i=1}^{n\_estimators}Y_{test}\left(i\right)$$

The KNN regression model involves considering three factors [40]: the number of nearest samples (*k*), the distance measurement method, and the regression prediction rule. The distance measurement method employs the widely used Euclidean distance, while the regression prediction rule is based on the mean method. The appropriate value for *k* can be determined through cross-validation based on the sample distribution. The missing value imputation process using KNN is outlined below.

Step 1: Calculate the Euclidean distance between the *s*-th sample $x_{test,s}$ in $X_{test}$ and the *t*-th sample $x_{train,t}$ in $X_{train}$, as shown in Formula (14). Then, calculate the distance between $x_{test,s}$ and all the mt samples in $X_{train}$ to obtain the distance vector $D\left(x_{test,s},\cdot \right)={\left[dist\left(x_{test,s},x_{train,1}\right),\;\ldots ,\;dist\left(x_{test,s},x_{train,mt}\right)\right]}^{T}$.

(14) $$dist\left(x_{test,s},x_{train,t}\right)=\sqrt{\sum_{i=1}^{n}{\left(x_{test,s_{i}}-x_{train,t_{i}}\right)}^{2}}$$

where $x_{test,s}(s=1,\ldots ,ms)$ is the *s*-th sample in $X_{test}$, $x_{train,t}(t=1,\ldots ,mt)$ is the *t*-th sample in $X_{train}$, *n* is the number of variables.

Step 2: Choose *k* nearest samples $\left[(x_{train,1}),\;\ldots ,\;(x_{train,k})\right]$ in $X_{train}$ according to the *k* smallest values in the distance vector $D(x_{test,s},\cdot )$.

Step 3: Calculate the average of the values $\left[(y_{train,1}),\;\ldots ,\;(y_{train,k})\right]$ in $Y_{train}$ that correspond to these *k* nearest samples, as shown in Formula (15), and set this average value $y_{s}$ as the predicted value for the sample $x_{test,s}$.

(15) $$y_{s}=\frac{1}{k}\sum_{i=1}^{k}\;\left(y_{train,i}\right)$$

Step 4: Repeat steps 1–3 to calculate predicted values for all samples in $X_{test}$, then all values in $Y_{test}$ are obtained.

(2) Imputation Process for Long-term Missing Variables

Since the multivariate regression model has the limitation that only one variable can be imputed in each process, an iterative method is designed to overcome this constraint. The iterative imputation based on the multivariate regression model can automatically complete the imputation of all long-term missing variables. The model from MLR, RF, or KNN is selected as multivariate regression model ${model}_{j}$. Assuming that $X_{k}^{\left(1\right)}\in R^{mk\times n}$ is the data segment after imputing transient isolated missing values, and $n_{long\_j}$ is the number of long-term missing variables in $X_{k}^{\left(1\right)}$. The iterative imputation based on a multivariate regression model is presented in Algorithm 1.
**Algorithm 1** The iterative imputation based on multivariate regression model**Input:** $X_{k}^{\left(1\right)}\in R^{mk\times n}$, $n_{long\_j}$**Output:** The imputed data segment $X_{k}^{\left(2\right)}\in R^{mk\times n}$
1. **Begin**2.Calculate the variable missing proportion ${VMP}_{j}$ for each long-term missing variable, and sort these variables in ascending order by ${VMP}_{j}$, get $\left\{\left(x_{\_\;,1}\right),(x_{\_\;,2}),\ldots ,\left(x_{{\_\;,n}_{long\_j}}\right)\right\}$;3. Set $X_{0}=X_{k}^{\left(1\right)}$;4. **For**
j=1 to $n_{long\_j}$:5.   Split $X_{j-1}$ into a training dataset $D_{train(j-1)}$ including only complete variables and a testing dataset $D_{test(j-1)}$ including only incomplete variables;6.   Train the multivariate regression model ${model}_{j}$ by inputting $X_{train(j-1)}$ formed by $n-n_{long\_j}+(j-1)$ complete variables from $D_{train(j-1)}$;7.   Input $X_{test(j-1)}$ formed by $n_{long\_j}-(j-1)$ incomplete variables from $D_{test(j-1)}$ into ${model}_{j}$, and get the predicted values $\left({\tilde{x}}_{\_\;,\;j}\right)$ for variable $\left(x_{\_,j}\right)$;8.    Impute $X_{j-1}$ using $\left({\tilde{x}}_{\_\;,\;j}\right)$;9.    Set $X_{j}=X_{j-1}$;10. **Return** $X_{k}^{\left(2\right)}=X_{n_{long\_j}}$;11. **End**

(3) Imputation Process for Short-term Missing Variables

The combination model based on single-dimensional interpolation and multivariate regression is developed for imputing the missing values of short-term missing variables. This combination model is based on the property that a missing variable experiences system fluctuations due to the influence of its related variables. The model utilizes a multivariate regression model to calculate the system fluctuation and incorporate it into the interpolation value. By considering the continuity in the time dimension and the correlation among different variables, this model significantly enhances imputation accuracy by combining the strengths of both models.

**Figure 2:**
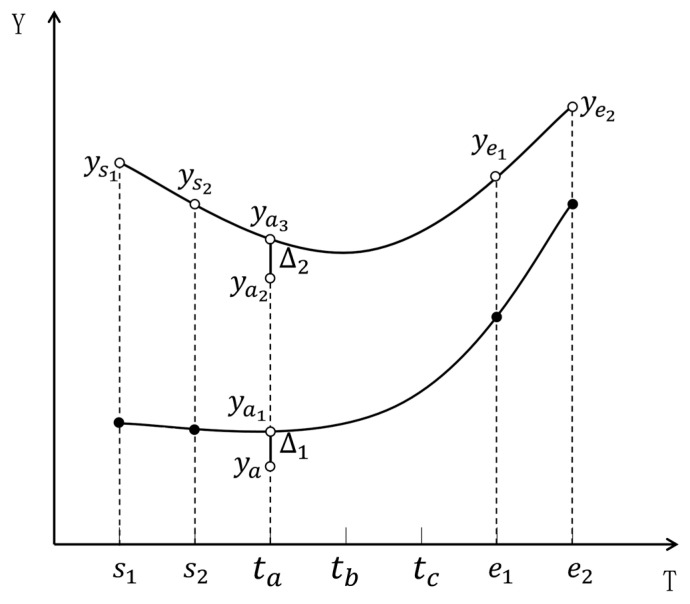
Example of short-term missing variable imputation based on the combination model.

**Figure 3:**
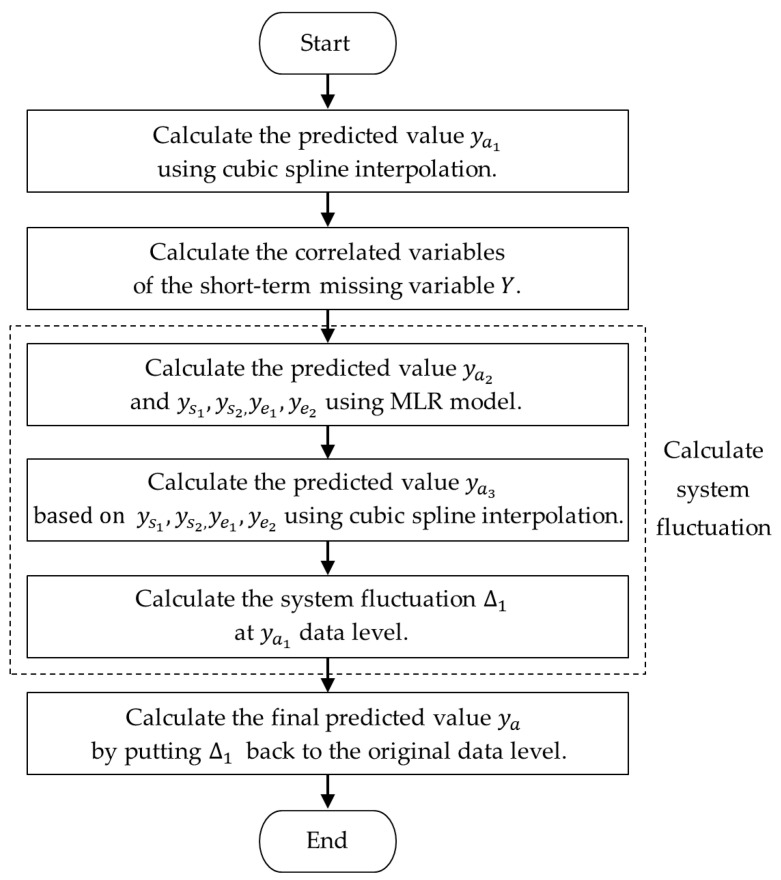
Imputation process for short-term missing variable based on the combination model.

Taking cubic spline interpolation and MLR as examples, the combination model for imputing missing values of short-term missing variables is designed. As shown in Figure 2, variable Y in data segment $X_{k}$ contains short-term missing from time $s_{2}$ to time $e_{1}$. $s_{1}$ and $e_{2}$, respectively, represent the corresponding time with a valid value on the left side of $s_{2}$ and on the right side of $e_{1}$. The continuous missing duration $∆t=\left|e_{1}-s_{2}\right|$, and ${Th}_{t1}<∆t\leq {Th}_{t2}$. Time $t_{a},t_{b},andt_{c}$ represent three sampling times in this period. $y_{a}$ represents the predicted value at time $t_{a}$, the imputation process for $y_{a}$ based on the combination model is shown in Figure 3.

Step 1: Calculate the predicted value $y_{a_{1}}$ at time $t_{a}$ using cubic spline interpolation by Formula (8).

Step 2: Calculate the correlation between variable X and the short-term missing variable Y using Formula (16). If $Cov(X,Y)>{Th}_{c}$, variable X is the correlated variable with Y. Then identify all the correlated variables with Y, denoted as $X_{j}(j=1,2,\ldots ,n_{c})$.

(16) $$Cov(X,Y)=\frac{\left(\sum_{i}^{mk}\left(x_{i}-\bar{x})(y_{i}-\bar{y}\right)\right)}{\sqrt{\sum_{i}^{mk}{\left(x_{i}-\bar{x}\right)}^{2}\sum_{i}^{mk}{\left(y_{i}-\bar{y}\right)}^{2}}}$$

where $\bar{x}=\frac{1}{mk}\sum_{i}^{mk}x_{i},\bar{y}=\frac{1}{mk}\sum_{i}^{mk}y_{i}$, mk is the sample size, ${Th}_{c}$ is the correlation threshold, $n_{c}$ is the number of correlated variables with Y.

Step 3: The variable Y is influenced by its correlated variables, which leads to system fluctuations. The MLR model and cubic spline interpolation are used to calculate the system fluctuation ${∆}_{1}$ at $y_{a_{1}}$ data level:

Firstly, use the MLR model for regression fitting to describe the relationship between Y and its correlated variables, and the corresponding predicted values $y_{s_{1}},\;y_{s_{2}},\;y_{e_{1}},\;y_{e_{2}},\;y_{a_{2}}$ at time $s_{1}{,\;s}_{2},\;e_{1},\;e_{2},\;t_{a}$ are calculated by Formula (12), where the dataset $X_{c\_y}\;\in R^{mj\times n_{c}}$ formed by all the correlated variables is used as the testing data, mj is the sample size of $X_{c\_y}$, and the sample size of $I_{test}$ in Formula (12) is set to mj.

Then construct a cubic spline interpolation function based on values $y_{s_{1}},\;y_{s_{2}},\;y_{e_{1}},\;y_{e_{2}}$ by Formula (8), and get the predicted value $y_{a_{3}}$ at time $t_{a}$.

Finally, calculate the system fluctuation ${∆}_{2}$ at $y_{a_{3}}$ data level by Formula (17). Since the system fluctuation is influenced by the data level, the relationship between ${∆}_{1}$ and ${∆}_{2}$ satisfies Formula (18). So the system fluctuation ${∆}_{1}$ is calculated by Formula (19).

(17) $${∆}_{2}=y_{a_{3}}-y_{a_{2}}$$

(18) $$\frac{{∆}_{1}}{y_{a_{1}}}=\frac{{∆}_{2}}{y_{a_{3}}}$$

(19) $${∆}_{1}=\frac{y_{a_{1}}}{y_{a_{3}}}{∆}_{2}$$

Step 4: Put the system fluctuation back to the original data level, as shown in Formula (20), and then the final predicted value $y_{a}$ at time $t_{a}$ is calculated:

(20) $$y_{a}=y_{a_{1}}-{∆}_{1}$$

#### 3.2.4. Continuous Missing Samples Imputation

After data splitting, the information between data segments is not only lost in time dimension but also among different variables. It is difficult to impute short-term and long-term missing samples using a single-dimensional interpolation model or a multivariate regression model. We adopt the LSTM model, which can effectively learn long-term dependencies, to impute continuous missing samples after imputing transient isolated missing values and continuous missing variables.

(1) LSTM Model

The 5-layer LSTM network for the prediction of missing values in continuous missing samples is as below.

Input layer: This layer receives input data, where the number of variables in the input data is consistent with the number of neurons in this layer.

**Figure 4:**
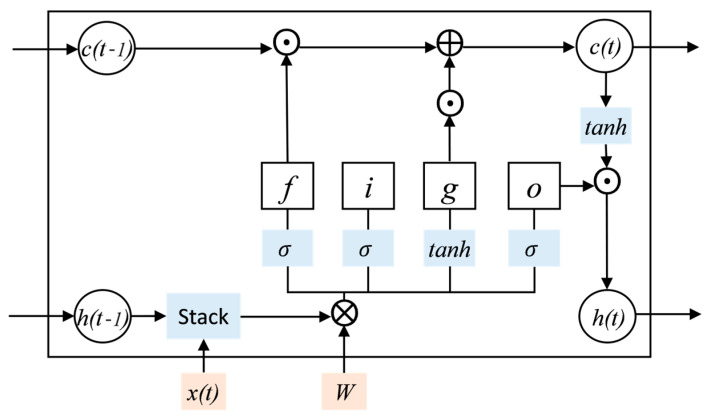
LSTM unit: *f* is the forget gate, *i* is the input gate, *g* is the update gate, *o* is the output gate, *c* is the unit state, *h* is the hidden state, σ is the activation function of Sigmoid, *W* is the weight matrix, Stack, ⊙, ⊕ and ⊗, respectively, represent matrix stacking, matrix elements multiplication, matrix addition and matrix multiplication.

LSTM layer: This layer builds the LSTM model. The LSTM unit structure is shown in Figure 4. The memory unit in LSTM has four gates: INPUT GATE (f), FORGET GATE (i), UPDATE GATE (g), and OUTPUT GATE (o). c(t) is the unit state, representing the information learned before time t, which can be seen as long-term memory. h(t) is the hidden state, representing the output of the network in the current state, which can be seen as short-term memory. x(t) is the current time network input value. The forget gate determines the retention degree of the current state c(t) to the cell state c(t−1) at the previous moment. The input gate determines the retention degree of the current state c(t) to the input x(t). The output gate controls the degree to which c(t) outputs to h(t) in the current state. Each node in the LSTM model can be calculated as below:

(21) $$i\left(t\right)=\sigma \left(W_{i}\cdot {\left[h\left(t-1\right),\;x\left(t\right)\right]}^{T}+b_{i}\right)\;f\left(t\right)=\sigma \left(W_{f}\cdot {\left[h\left(t-1\right),\;x\left(t\right)\right]}^{T}+b_{f}\right)\;o\left(t\right)=\sigma \left(W_{o}\cdot {\left[h\left(t-1\right),\;x\left(t\right)\right]}^{T}+b_{o}\right)\;g\left(t\right)=\tanh\left(W_{g}\cdot {\left[h\left(t-1\right),\;x\left(t\right)\right]}^{T}+b_{g}\right)\;c\left(t\right)=f\left(t\right)⊙c\left(t-1\right)+i\left(t\right)⊙g\left(t\right)\;h\left(t\right)=o\left(t\right)⊙\tanh\left(c\left(t\right)\right)$$

where *f* is the forget gate, *i* is the input gate, *g* is the update gate, *o* is the output gate, *c* is the unit state, *h* is the hidden state, *σ* is the activation function of Sigmoid, *W* is the weight matrix, *b* is the bias term, ⊙ represent matrix elements multiplication.

Lost layer: This layer is used to prevent overfitting [41]. During the training process, the loss probability $P_{lost}$ is set to 0.5. The input data from the LSTM layer is randomly set to 0 with rate $P_{lost}$. The remaining data is scaled by the rate $1/(1-P_{lost})$ and then input into the fully connected layer.

Fully connected layer: This layer establishes full connection between the LSTM layer with the output layer. The number of input neurons in this layer is equal to the number of neurons in LSTM layer.

Output layer: This layer generates the prediction results. The number of output neurons is equal to the number of variables in the output data.

(2) Imputation Process for Continuous Missing Samples

The LSTM model takes all the complete data segments before the current moment as input and predicts the missing values at the current moment. Then the imputed values are used as input to predict the missing values at the next moment. Therefore, the continuous missing samples (the missing data segments) are imputed by iteratively executing the model. The iterative imputation process for the missing data segment $X_{k^{*}}^{*}\in R^{mc\times n}$ is as follows, where *mc* is the sample size and *n* is the number of variables. And *l* represents the time steps (the length of input data) of the LSTM model.

Step 1: LSTM model training.

Step 1.1: Generate the training dataset based on data segment ${X_{k}}^{\left(2\right)}\in R^{mk\times n}$ after imputing all transient isolated missing values and continuous missing variables.

Step 1.2: Initialize i=1 and train input data, where the *i*-th input sample of $X_{train}$ is $X_{train,\;i}=[x_{train,i},\;\ldots ,\;x_{train,i+l-1}]$; then train output data, where the *i*-th output sample of $X_{test}$ is $X_{test,\;i}=x_{test,i+l}$.

Step 1.3: Repeat Step 1.2 mk−l times.

Step 1.4: Train the LSTM model ${model}_{LSTM}$ based on dataset $X_{train}$ and $X_{test}$, then get the trained LSTM model ${model}_{LSTM\_h(0)}$ whose output state is h(0).

Step 2: Missing data prediction.

Step 2.1: Initialize the LSTM model, and input the training dataset $X_{train}$ into ${model}_{LSTM\_h(0)}$ to obtain ${model}_{LSTM\_h(mk)}$ whose output state is h(mk).

Step 2.2: For t=mk+1, input the *l* consecutive samples before time *t* (i.e., $X_{train,\;t-1}=[x_{train,mk-l+1},\;\ldots ,\;x_{train,mk}]$) into ${model}_{LSTM\_h(t-1)}$ to obtain the predicted data ${\tilde{x}}_{t}$. Then update ${model}_{LSTM\_h(t-1)}$ according to Formula (23) and get ${model}_{LSTM\_h(t)}$.

Step 2.3: Repeat Step 2.2 until t=mk+mc, then get the predicted data segment ${X_{k^{*}}^{*}}^{\left(1\right)}=[{\tilde{x}}_{mk+1},\ldots ,\;{\tilde{x}}_{mk+mc}]$.

It should be noted that the input of the LSTM model is a vector, so it is necessary to reconstruct the data matrix into a vector before model training and prediction.

### 3.3. The Hybrid Missing Data Imputation Method

Considering the various types and high missing proportion of missing data in batch process monitoring datasets, we propose a hybrid missing data imputation method based on the above research. The method classifies missing data according to the predefined classification rules, then combines and improves a single-dimensional interpolation model, a multivariate regression model, and LSTM to step-by-step impute different categories of missing data based on their specific characteristics. The pseudocode of this hybrid method is presented in Algorithm 2.
**Algorithm 2** The proposed hybrid missing data imputation method**Input:** The original dataset $X_{ORG}$
**Output:** The imputed complete dataset $X_{IMP}$
1. **Begin**2. **Unfolding** data along the batch dimension, get the 2D dataset *X*;3.**Classifying** the missing data into five categories: transient isolated missing values, short-term missing variables, long-term missing variables, short-term missing samples and long-term missing samples;4. **Splitting** dataset *X*, get $X=[X_{1},\;X_{1}^{*},\;\ldots ,X_{k},\;X_{k^{*}}^{*},\;\ldots ,\;X_{K-1},X_{K-1}^{*},\;X_{K}]$;5.**Imputing transient isolated missing values** in each data segment $X_{k}$ using single-dimensional interpolation models; 6. ${X_{k}}^{\left(1\right)}(k=1,\;...,\;K)$ ← The imputed data segments;7. **Standardize** each data segment;8.**Imputing long-term missing variables** in each data segment $X_{k}$ using the iterative imputation based on multivariate regression model, and **imputing short-term missing variables** in each data segment $X_{k}$ using the combination model based on single-dimensional interpolation and multivariate regression;9. ${X_{k}}^{\left(2\right)}(k=1,\;...,\;K)$ ← The imputed data segments;10.**Imputing short-term missing samples and long-term missing samples** (i.e., the missing data segments $X_{k^{*}}^{*}$) using LSTM model;11. ${X_{k^{*}}^{*}}^{\left(1\right)}(k^{*}=1,\;\ldots ,\;K-1)$ ← The imputed data segments;12. Complete dataset $X_{IMP}$ ← De-standardize, and transform 2D data to 3D data;13. **End**

**Figure 5:**
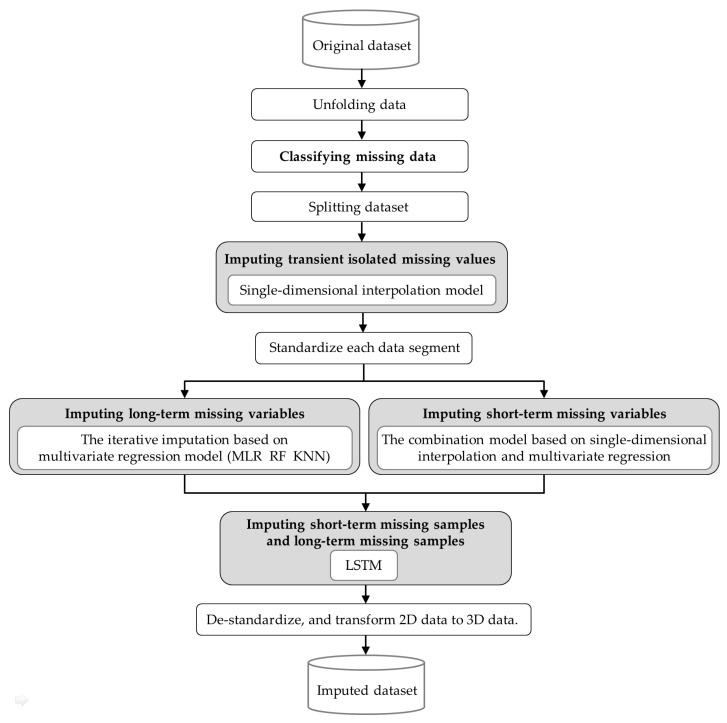
The proposed hybrid missing data imputation method.

As shown in Figure 5, the proposed hybrid missing data imputation method consists of the following eight steps:

Step 1: Unfolding data: The original three-dimensional dataset $X_{ORG}$ is unfolded along the batch dimension to obtain two-dimensional dataset *X*.

Step 2: Classifying missing data: According to the missing data classification method (Section 3.1.2), the continuous missing duration ∆t for each variable and the corresponding number of variables $n_{v}$ missing simultaneously are calculated. By comparing the calculated results with the threshold values, the missing data are classified into five categories: transient isolated missing values, short-term missing variables, long-term missing variables, short-term missing samples, and long-term missing samples.

Step 3: Splitting dataset: The dataset X is split according to the locations of continuous missing samples, then get $X=[X_{1},\;X_{1}^{*},\;\ldots ,X_{k},\;X_{k^{*}}^{*},\;\ldots ,\;{X_{K-1},X}_{K-1}^{*},\;X_{K}]$, where $X_{k}(k=1,\;\ldots ,\;K)$ represents the *k*-th data segment (the data segment with transient isolated missing values, short-term or long-term missing variables), $X_{k^{*}}^{*}(k^{*}=1,\;\ldots ,\;K-1)$ represents the missing data segment (the data segment with short-term or long-term missing samples) between the *k*-th and (*k +* 1)-th data segments.

Step 4: Imputing transient isolated missing values: Transient isolated missing values in each data segment $X_{k}$ are imputed using three single-dimensional interpolation models as mentioned in Section 3.2.2, and the corresponding imputed data segments are ${X_{k}}^{\left(1\right)}(k=1,\;\ldots ,\;K)$.

Step 5: Standardize each data segment $X_{k}$: Taking the variable *j* in data segment $X_{k}$ as an example, values are standardized using z-score standardization:

(22) $$x_{i,\;j}^{z}=\left(x_{i,j}-\mu_{j}\right)/\sigma_{j}$$

where $x_{i,\;j}^{z}$ is the standardized value of the *i*-th sample $x_{i,j}(i=1,\;\ldots ,\;mk,\;j=1,\;\ldots ,\;n)$, $\mu_{j}$ is the mean of variable *j*, $\sigma_{j}$ is the standard deviation of variable *j*, mk and *n,* respectively, represent the sample size and the number of variables in $X_{k}$.

Step 6: Imputing long-term missing variables and short-term missing variables: For each data segment ${X_{k}}^{\left(1\right)}$, each long-term missing variable are imputed using the iterative imputation based on multivariate regression model as mentioned in Section 3.2.3 (2), all short-term missing variables are imputed using the combination model based on single-dimensional interpolation and multivariate regression as mentioned in Section 3.2.3 (3), and the corresponding imputed data segments are ${X_{k}}^{\left(2\right)}(k=1,\;\ldots ,\;K)$.

Step 7: Imputing short-term missing samples and long-term missing samples (i.e., the missing data segments): Taking ${X_{k}}^{\left(2\right)}(k=1,\;\ldots ,\;K)$ as input, all missing data segments $X_{k^{*}}^{*}$ are imputed using LSTM model as mentioned in Section 3.2.4, and the corresponding imputed data segments are ${X_{k^{*}}^{*}}^{\left(1\right)}(k^{*}=1,\;\ldots ,\;K-1)$.

Step 8: De-standardize the imputed data segments and transform two-dimensional data to three-dimensional data, then get the imputed complete dataset $X_{IMP}$.

## 4. Illustration and Discussion

### 4.1. Data Source and Description

Injection molding, which refers to the process of making semi-finished parts of a certain shape from molten raw materials, is a typical batch process. A publicly accessible real-world injection molding dataset [42] is taken as an example, which contains data collected from both mold temperature control machines and mold sensors. Six process variables are selected, as shown in Table 2. Under this operating condition, a total of 100 normal batches with 919 sampling points are obtained, denoted as $X_{ORG}(100\times 6\times 919)$. The dataset needs to be unfolded along the batch dimension to obtain two-dimensional dataset X(6×91,900). It includes six variables, and the length of each variable is 91,900 sampling points. The dataset contains data fluctuations, repeating trends between different batches, and dynamic correlations among different variables.

### 4.2. Performance Evaluation Index

(1) Root Mean Square Error

To measure the missing data imputation accuracy, we adopt the most commonly used measure in this field: Root Mean Square Error (*RMSE*) [19]. The *RMSE* index can reflect the deviation between the predicted value and the actual value. The smaller the value of *RMSE*, the higher the accuracy of the algorithm. Taking variable *j* as an example, the *RMSE* value can be calculated as follows:

(23) $${RMSE}_{j}=\sqrt{\frac{1}{n_{j}}\sum_{i=1}^{n_{j}}{\left(x_{i,j}-{\tilde{x}}_{i,j}\right)}^{2}}$$

where $n_{j}$ is the number of missing values of variable *j* in data segment $X_{k}$, $x_{i,j}$ is the actual value, ${\tilde{x}}_{i,j}$ is the predicted value of $x_{i,j}$.

(2) Mean Square Error

The performance of KNN, RF, and LSTM models for missing value prediction depends on the selection of hyperparameters. We adopt Mean Square Error (*MSE*) to construct the loss function and utilize 10-fold cross-validation to determine the optimal hyperparameters. The smaller the value of *MSE*, the higher the accuracy of the algorithm. The *MSE* value can be calculated as follows:

(24) $$MSE=\frac{1}{m\times n}\sum_{i=1}^{m}\sum_{j=1}^{n}{\left(x_{i,j}-{\tilde{x}}_{i,j}\right)}^{2}$$

where *m* is the sample size, *n* is the number of variables, $x_{i,j}$ is the actual value, ${\tilde{x}}_{i,j}$ is the predicted value of $x_{i,j}$.

### 4.3. Data Processing

Firstly, the original three-dimensional dataset $X_{ORG}(100\times 6\times 919)$ was unfolded along the batch dimension to obtain a two-dimensional dataset X(6×91,900). According to the missing data classification rules defined in Section 3.1.2, the categories of missing data were determined. The dataset X contains two data segments with continuous missing samples. Therefore, it was split into three data segments and two missing data segments according to the locations of continuous missing samples, i.e., $X={\left[X_{1},\;X_{1}^{*},X_{2},\;X_{2}^{*},X_{3}\right]}^{T}$. Data segments $X_{1},\;X_{2},\;X_{3}$ contain transient isolated missing values and continuous missing variables, while the two missing data segments $X_{1}^{*},X_{2}^{*}$ are the data segments with continuous missing samples. In data segment $X_{1}$, the plasticizing pressure variable contains continuous missing, while the cylinder pressure and SV2 value opening variables contain transient isolated missing values. In data segment $X_{2}$, all variables only contain transient isolated missing values. In data segment $X_{3}$, the plasticizing pressure variable contains continuous missing, while the nozzle temperature, cylinder pressure and SV2 value opening variables contain transient isolated missing values. The data integrity information is presented in Table 3. Considering the missing proportions of six process variables, we selected data segment $X_{2}$ with the lowest missing proportion to evaluate transient isolated missing value imputation and utilized the plasticizing pressure variable with continuous missing in data segment $X_{1}$ to evaluate continuous missing variable imputation.

### 4.4. Missing Data Imputation and Results Analysis

#### 4.4.1. Transient Isolated Missing Values Imputation

In order to better compare the performance of different imputation methods, some transient isolated values in data segment $X_{2}$ were randomly deleted to obtain four experimental datasets with missing proportions of 5%, 10%, 15%, and 20%. The detailed imputation process for transient isolated missing values is shown in Section 3.2.2. The mean and hot-desk imputation methods were selected as baseline models.

The RMSE values for the predicted values of the six process variables calculated are shown in Table 4. Experimental results show that the single-dimensional interpolation model performs better than the mean and hot-desk imputation methods. This difference becomes more pronounced with an increasing proportion of missing values. When the missing proportion reaches 20%, the RMSE value of the single-dimensional interpolation model for the screw speed variable is 1.129, which is only about 1/3 of that obtained with the mean method.

#### 4.4.2. Continuous Missing Variables Imputation

To evaluate the performance of different imputation methods for imputing the continuous missing variable, the continuous missing variable (plasticizing pressure) in the data segment $X_{1}$ was imputed. The transient isolated missing values in data segment $X_{1}$ were imputed first. Methods based on single-dimensional interpolation model and a multivariate regression model were used for imputation. The detailed imputation process for the continuous missing variable is shown in Section 3.2.3.

(1) Hyperparameters Selection

**Figure 6:**
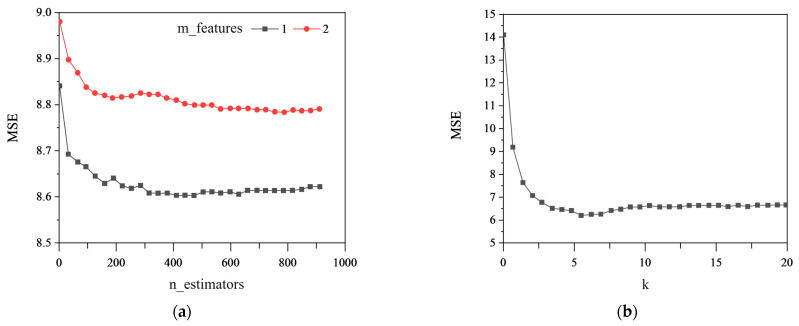
Hyperparameter selection through 10-fold cross-validation: (**a**) n_estimators and m_features of the RF model; (**b**) *k* of the KNN model.

The hyperparameters of the RF and KNN models were selected through 10-fold cross-validation, and the results are presented in Figure 6. Figure 6a shows that the optimal parameters n_estimators and m_features for the RF model are suitable to select 500 and 1, where n_estimators is the number of CART decision trees and m_features is the number of selected features. Figure 6b shows that the optimal parameter *k* for the KNN model is suitable for selecting 7, where *k* is the number of nearest samples.

(2) Imputation Results Analysis

The combination model based on single-dimensional interpolation and multivariate regression was utilized for imputation, while six baseline models were employed for comparison. The RMSE values calculated using different methods are presented in Table 5. Experimental results show that the multivariate regression model performs better than the single-dimensional interpolation model. And the combination of a single-dimensional interpolation model and a multivariate regression model exhibits improved imputation accuracy. In particular, the combination of single-dimensional interpolation and MLR achieves the highest imputation accuracy, with an RMSE value of only 1.976. This further indicates the significance of considering both the continuity in the time dimension and the correlation between variables when dealing with short-term missing variables.

#### 4.4.3. Continuous Missing Samples Imputation

In order to evaluate the imputation accuracy of short-term and long-term missing samples, the missing data segments $X_{1}^{*}$ and $X_{2}^{*}$ were imputed based on LSTM model after completing the imputation of all transient isolated missing values and continuous missing variables in data segments $X_{1}$, $X_{2}$ and $X_{3}$. The detailed imputation process for continuous missing sample is shown in Section 3.2.4.

(1) Hyperparameters Selection

The parameters Lr and l have a significant impact on the performance of LSTM, where Lr represents the learning rate and l represents the time steps. They were optimized separately, considering their minimal mutual influence. Initially, the LSTM network was initialized with the following parameters: the number of neurons was set to 120, the number of iterations was set to 400, the Adam optimization algorithm was used as the Optimizer, a gradient threshold of 1 was set to prevent gradient explosions, and the dropout rate $P_{lost}$ was set to 0.

**Figure 7:**
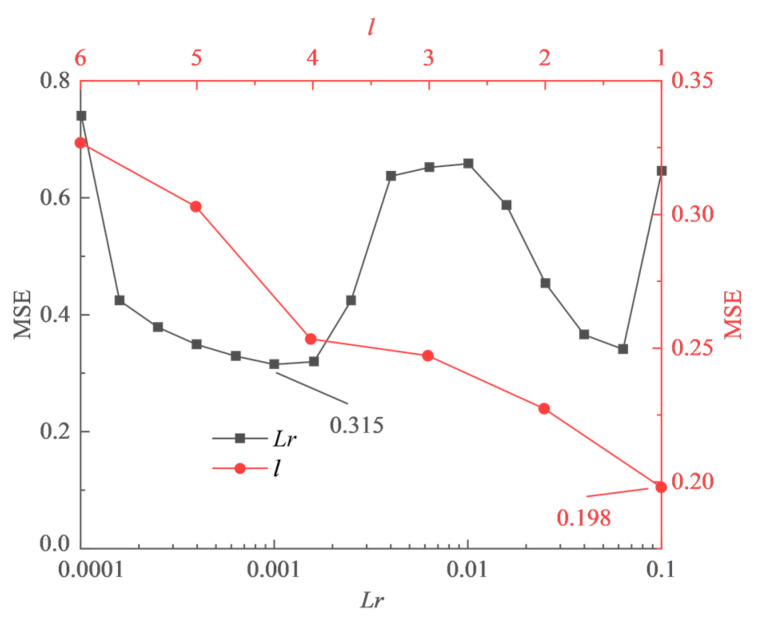
Hyperparameters selection for LSTM model through 10-fold cross-validation.

The parameters Lr and l were selected through 10-fold cross-validation. For Lr, the early stopping technique was applied to prevent overfitting. The frequency of verification was set to 20, and the tolerance of verification was set to 4. While for l, the dropout rate was set to 0.2 as a replacement for the early stopping technique to prevent overfitting. The results obtained for parameters Lr and l through 10-fold cross-validation are shown in Figure 7.

As Lr is increased from 0.0001 to 0.1, the MSE curve initially exhibits a rise followed by a decline. However, when Lr exceeds 0.1, training begins to fail. Therefore, Lr is set to 0.001. The MSE curve for l shows an almost linearly increasing trend, which indicates that the imputation accuracy will decrease as the historical input data increases. Therefore, l is set to 1. In addition, when using the lost layer instead of the early stopping technique to prevent overfitting, the MSE value decreases from 0.315 to 0.198. This indicates that the lost layer is more effective in preventing overfitting than the early stopping technique.

(2) Imputation Results Analysis

The ARIMA (Autoregressive Integrated Moving Average) [43,44] and ELM [36,45] were selected as baseline models. ARIMA is a classical time series model that combines autoregressive, differencing, and moving average components to predict missing values through data autocorrelation. ELM is an efficient machine learning model based on a single-layer feedforward neural network that uses multiple features to predict missing values. The number of hidden layer neurons of both the ELM and LSTM models is the same. The RMSE values calculated for different models are shown in Table 6. The results show that the LSTM model exhibits higher imputation accuracy compared to the ARIMA and ELM models. This indicates that the LSTM model is more effective in capturing long-term dependencies in time series data.

## 5. Conclusions

In real-world batch process monitoring datasets, missing data usually occurs in different patterns. Failing to identify the type of missing data or applying imputation methods regardless of the missing type may decrease imputation performance. Many imputation methods have been developed to impute the missing data; however, most of them still do not fulfill the need for data quality in datasets with different types of missing data. Therefore, this paper proposes a novel hybrid missing data imputation method to deal with different types of missing data in a real-world batch process monitoring dataset. By classifying missing data into five distinct categories, we combine and improve suitable models to step-by-step impute different categories of missing data based on their unique characteristics. Through experiments taking a real-world injection molding process monitoring dataset as an example, it can be concluded that missing data pattern analysis combined with appropriate models to impute missing data has better imputation accuracy. Therefore, the hybrid method proposed in this paper excels at missing data imputation for complex batch process monitoring datasets. In practical applications, this method can be employed to impute missing data in batch process monitoring datasets, and the design concept of first categorizing and then stepwise imputing based on data features in this method can also be extended to other datasets containing different types of missing data.

In future research, we plan to conduct studies on the following aspects: The 10-fold cross-validation method, employed for hyperparameter selection in LSTM models, still needs some degree of manual tuning; Bayesian Optimization or Successive Halving could be introduced for automated optimization. Although we have designed a missing data classification method, automated techniques for missing data classification need to be further explored. Data noise can potentially impact imputation performance, and methods such as data cleaning or outlier detection to preprocess the data for noise elimination can be explored. Furthermore, referring to the benchmark proposed in reference [19], additional metrics besides RMSE, such as MAE and runtime, can be introduced. A comprehensive evaluation of imputation accuracy and efficiency could be conducted by selecting suitable baseline methods and utilizing multiple batch process monitoring datasets, while considering various factors like the missing block size, the number of sequences, etc. Based on the evaluation results, the proposed hybrid method might be further improved by enhancing existing models or introducing new models.

## Figures and Tables

**Table 1:** Classification rules for missing data in batch process monitoring dataset.

Missing Data Categories	Classification Rules
Transient isolated missing values	$T_{0}\leq ∆t\leq {Th}_{t1}$
Short-term missing variables	${Th}_{t1}<∆t\leq {Th}_{t2}$ and $n_{v}<{Th}_{v}$
Long-term missing variables	$∆t>{Th}_{t2}$ and $n_{v}<{Th}_{v}$
Short-term missing samples	${Th}_{t1}<∆t\leq {Th}_{t2}$ and $n_{v}\geq {Th}_{v}$
Long-term missing samples	$∆t>{Th}_{t2}$ and $n_{v}\geq {Th}_{v}$

**Table 2:** Process variables of a real-world injection molding process monitoring dataset.

Variable Type	Variable Description	Unit
Process	Screw speed	Mm/s
	Plasticizing pressure	Bar
	Nozzle temperature	℃
	Cylinder pressure	Bar
	SV1 value opening	%
	SV2 value opening	%

**Table 3:** Data integrity information.

Data Segment	$X_{1}$	$X_{2}$	$X_{3}$
SMP(k)	0.216	0.037	0.130
Screw speed ${VMP}_{1}\left(k\right)$	0	0.004	0
Plasticizing pressure ${VMP}_{2}(k)$	0.215	0.029	0.129
Nozzle temperature ${VMP}_{3}(k)$	0	0	0.002
Cylinder pressure ${VMP}_{4}(k)$	0.002	0.002	0.003
SV1 value opening ${VMP}_{5}(k)$	0	0	0
SV2 value opening ${VMP}_{6}(k)$	0.029	0.017	0.003

**Table 4:** RMSE of missing data imputation results for transient isolated missing values.

Imputation Method	$X_{2}$	Screw Speed	Plasticizing Pressure	Nozzle Temperature	Cylinder Pressure	SV1 Value Opening	SV2 Value Opening
Single-dimensional interpolation model	5%	1.051	2.056	3.881	2.089	0.103	0.893
Mean		2.673	3.385	4.532	2.053	0.067	1.426
Hot-deck imputation		1.105	2.734	4.364	2.047	0.032	1.940
Single-dimensional interpolation model	10%	1.438	2.072	3.659	2.078	0.056	0.912
Mean		2.937	3.619	4.233	2.058	0.099	1.503
Hot-deck imputation		1.935	2.802	4.674	2.049	0.042	1.784
Single-dimensional interpolation model	15%	1.301	2.089	3.431	2.067	0.055	1.425
Mean		3.801	3.623	4.567	2.108	0.112	1.285
Hot-deck imputation		2.572	2.723	4.347	2.087	0.045	1.731
Single-dimensional interpolation model	20%	1.129	2.078	3.626	2.074	0.054	1.373
Mean		3.256	3.611	4.910	2.099	0.113	1.891
Hot-deck imputation		2.533	2.805	4.221	2.072	0.051	1.992

**Table 5:** RMSE of missing data imputation results for continuous missing variables.

Imputation Method		RMSE
The combination model based on single-dimensional interpolation and multivariate regression model	Single-dimensional interpolation + MLR	1.976
	Single-dimensional interpolation + RF	2.016
	Single-dimensional interpolation + KNN	2.159
Single-dimensional interpolation model	Linear interpolation	5.812
	Mean	6.031
	Spline interpolation	5.903
Multivariate regression model	MLR	4.392
	RF	4.204
	KNN	4.450

**Table 6:** RMSE of missing data imputation results for continuous missing samples.

Imputation Method	Missing Data Segment	Screw Speed	Plasticizing Pressure	Nozzle Temperature	Cylinder Pressure	SV1 Value Opening	SV2 Value Opening
LSTM	$X_{1}^{*}$	0.842	1.098	2.719	1.093	0.112	0.149
ARIMA		1.691	1.104	2.903	1.007	0.119	0.201
ELM		2.715	1.124	2.812	1.132	0.105	0.218
LSTM	$X_{2}^{*}$	0.529	1.071	2.027	1.073	0.094	0.173
ARIMA		1.626	1.176	2.297	1.519	0.113	0.191
ELM		2.371	1.193	2.151	1.168	0.151	0.264

## Data Availability

Not applicable.

## Abbreviations

LSTM	Long Short-Term Memory
LR	Linear Regression
MLR	Multivariate Linear Regression
SVD	Singular Value Decomposition
PCA	Principal Component Analysis
MF	Matrix Factorization
CD	Centroid Decomposition
EM	Expectation Maximization
KNN	K Nearest Neighbor
RF	Random Forest
ELM	Extreme Learning Machine
RNNs	Recurrent Neural Networks
VMP	Variable Missing Proportion
SMP	Sample Missing Proportion
CART	Classification and Regression Tree
RMSE	Root Mean Square Error
MSE	Mean Square Error
ARIMA	Autoregressive Integrated Moving Average
